# Sustained accuracy improvement in intraocular lens power calculation with the application of quality control circle

**DOI:** 10.1038/s41598-017-14171-9

**Published:** 2017-11-01

**Authors:** Lei Lin, Pingjun Chang, Jialu Xie, Zhangliang Li, Hongfang Zhang, Fan Lu, Yun-e Zhao

**Affiliations:** School of Ophthalmology and Optometry, Eye Hospital, Wenzhou Medical University, Wenzhou, Zhejiang, China

## Abstract

Accurate intraocular lens (IOL) power calculation is always a challenge in ophthalmology, and unoptimized process may lead to inaccurate refractive outcomes. Quality control circle (QCC) has shown its success in many fields as a process management tool. However, its efficacy in ophthalmology remains unclear. Here we utilized the QCC method to optimize the process and evaluate its efficacy in improving the accuracy of IOL power calculation. After the QCC application, the percentage of eyes with achieved refractive outcomes within 0.5 diopter significantly increased from 63.2% to 80.8% calculated by Haigis formula and 59.2% to 75.8% by SRK/T formula in patients with normal axial length (AL) (22 mm ≤ AL < 26 mm). Although there were no statistically significant differences in patients with long AL by the two formulas (p = 0.886 and 0.726), we achieved an accuracy of 75% with the application of the PhacoOptics software, which was significantly higher than that using the other two formulas (p < 0.001). Our findings indicated that QCC optimized and standardized the process of IOL power calculation, thus improved the accuracy of IOL power calculation in patients who underwent cataract surgery.

## Introduction

With the remarkable update of surgical technology and equipment, phacoemulsification combined with intraocular lens (IOL) implantation has become the most prevalent treatment for cataract. Although precision medicine is becoming an appealing concept, accurate IOL power calculation is always a challenge in ophthalmology, while the involvement of various departments, doctors and technicians made it more complicated^1–9^.

In many hospitals, the technicians performed the preoperative examinations on cataract patients including measurement of axial length, anterior chamber depth and corneal curvature. Then according to the results of biometric instrument IOL-Master or Lenstar (A-scan results were utilized in patients who were unable to be measured by biometric instrument), the surgeon selected the formula, calculated the IOL power and postoperative refractive prediction. The final refractive results were finished by optometrists. Hence, every simple step during the process may cause errors, and unoptimized process will inevitably result in undesirable postoperative refractive outcomes^1,5,10,11^.

As a process management and problem-solving technique, quality control circle (QCC) was firstly employed in business management and company operation in Japan^12–15^. With the joint effort by all members and referring to certain procedures, the group members can make the most of their advantages and cooperate with other related departments, thus finally improving the complex work flow and solve the problems in work and management^13,14,16^. Some studies evaluated the role of QCC in medical improvement and highlighted the success of its application in medical and hospital management^17–19^. However, to our knowledge, little literature focused on the unoptimized process of IOL power calculation and the application of QCC in the field of cataract. Therefore, the aim of this study was to apply the novel QCC method to optimize the IOL power calculation process under the QCC principle and evaluate its accuracy and efficacy in accuracy improvement.

## Materials and Methods

### Patients

This study was approved by the Institutional Review Board of the Eye Hospital of Wenzhou Medical University and written informed consents were obtained from all patients. This study was complied with the tenets of the Declaration of Helsinki and all methods were performed in accordance with relevant protocol. Retrospective data of 116 patients (170 eyes) who had undergone phacoemulsification and IOL implantation between November 2013 and September 2014 at our hospital was included (Without QCC group). After applying the optimized process assisted by QCC, 104 patients (151 eyes) that underwent the surgery by the same experienced surgeon (Yun-e Zhao) between March 2015 and January 2016 were regarded as the QCC group. We excluded eyes with pterygium, strabimus, epiretinal membrane, retinal detachment, uveitis, or a history of corneal or intraocular surgery. Patients with complications or a best corrected visual acuity less than 20/40 after surgery were also excluded. According to the axial length (AL), patients were divided into the normal AL (22 mm ≤ AL < 26 mm) and long AL (AL ≥ 26 mm) subgroups.

### Process optimization and standardization

**Figure 1:**
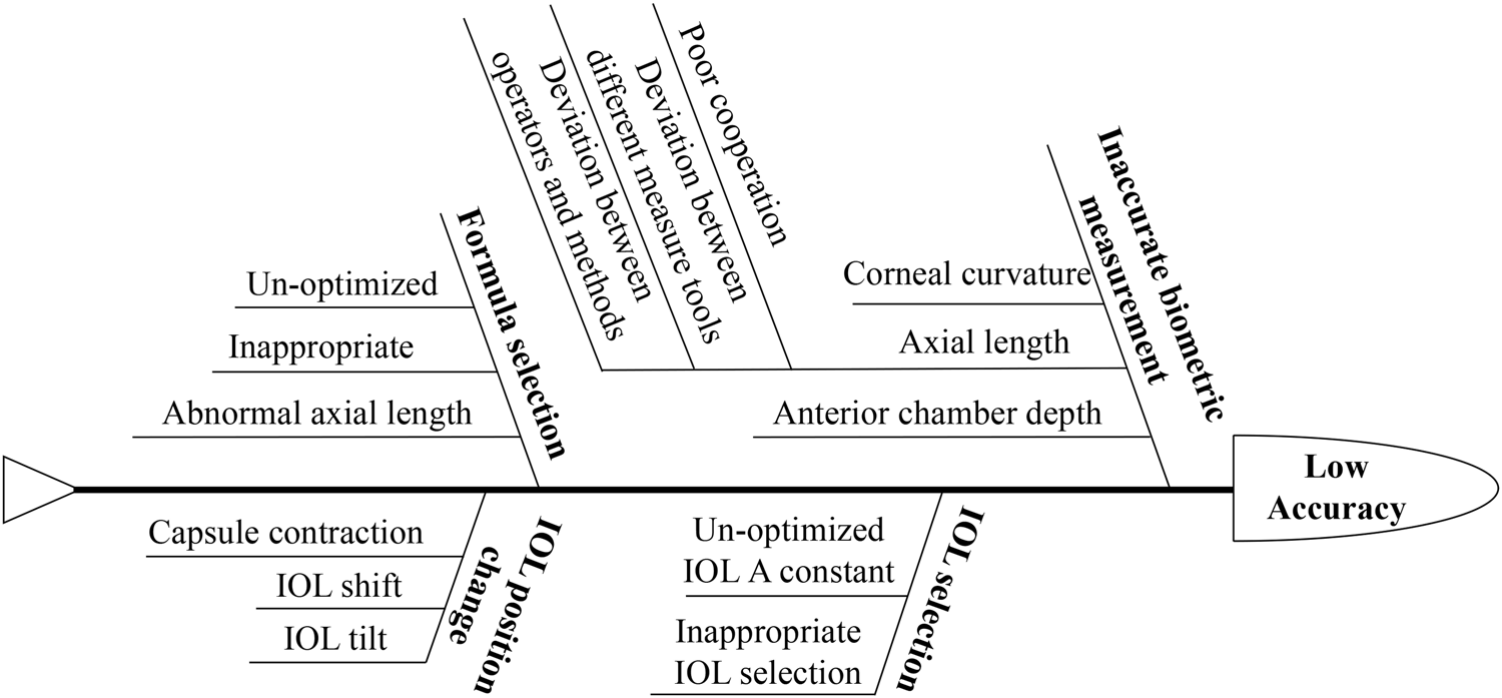
Fishbone analytical diagram. This fishbone diagram includes main reasons that lead to the low accuracy of intraocular lens (IOL) power prediction.

The QCC technique was applied to optimize and standardize the process of IOL power calculation. The retrospective data of 117 patients were reviewed to analyze the potential reasons based on fishbone analytical diagram method (Fig. 1). Then every QCC members voted to choose the main target. Finally, three leading causes were selected as the main target of this QCC (Table 1), which were respectively inaccurate biometric measurement, IOL and formula selection and optimization. Taking the feasibility, economy and circle capacity into consideration, two experienced doctors from the cataract department and 1 technician participated in the strategy assessment and graded each strategy based on a 5-point system. The strategy with the lowest score would not be selected in this study. According to the main reasons, sufficient circle meetings were hold to optimize the positive strategies as follows.

**Table 1:** Reason analysis and strategy assessment.

Reason analysis	Strategy	Assessment			G	Selection (Y/N)
		F	E	C		
A. Inaccurate measurement of axial length	A1. Repeated measurement and choose the average value/performed by different technicians	15	14	15	44	Y
	A2. Performed under B scan-guidance (for A scan)	14	15	13	42	Y
B. Inaccurate measurement of corneal curvature and ACD	B1. Standard protocol and compare the results by different technicians	15	14	15	44	Y
C. Formula selection and optimization	C1. Two formula optimizations of different IOL types	15	15	15	45	Y
	C2. Formula optimization for special ACD or K values	13	12	12	37	N
	C3. Application of the PhacoOptics software	15	14	14	43	Y

F = Feasibility, E = Economy, C = Circle capacity, G = Grade, ACD = Anterior chamber depth, IOL = intraocular lens, Y = Yes, N = No. The strategy assessment was graded by two experienced doctors and 1 technician based on a 5-point system.

Firstly, normalize the biometric measure protocol for IOLMaster: (1) make sure that the examined eye of patients focused on the fixation lamp and use penlight to guide the fellow eye if the fixation is poor; (2) delete the data with a deviation of more than 0.02 mm, and reexamine the data less than 5 groups; while the most important tip for curvature measurement is nice focus; then the notes for the A scan: (a) adjust the sonic velocity related to lens opacity; (b) ensure the favorable fixation of the patients with the red dot; (c) determine and record the final data based on the adjusted waveform in A scan, delete the data with an abnormal waveform or a deviation of more than 0.05 mm from the mean value, reexamine the data less than 10 groups, and assign another technician to finish the examination if the measurement results could not meet the standard mentioned above.

Secondly, optimize the IOL constant in the light of User Group for Laser Interference Biometry (ULIB, website: http://ocusoft.de/ulib/). Thirdly, select the ray-tracing assisted IOL calculation software PhacoOptics which was reported by Olsen when it comes to patients with long AL^20,21^. During the QCC activity, the quality management cycle, namely PDCA (plan, do, check and action), should be followed throughly to achieve the sustained enhancement of strategy execution.

### Main outcome measurement

The results of the subjective refraction at 3 months postoperatively were recorded as achieved refraction and expressed as the spherical equivalent (SE). The predicted refraction error was defined as the achieved refraction minus the predicted refraction result by each formula. The mean difference and absolute difference of the predicted refraction were calculated as the mean prediction error (ME) and mean absolute prediction error (MAE), respectively. Additionally, the percentage of eyes with a final MAE within 0.5D was regarded as an evaluation index of the accuracy.

### Statistical analyses

The sample size in the QCC group was determined by PASS 11.0 software (NCSS Statistical Software, Kaysville, UT) based on 80% power to detect the effect of QCC. Then the data was analyzed by SPSS 18.0 software for Windows (SPSS Inc. Chicago, IL, U.S.). Normal distribution of the data was analyzed by the Kolmogorov-Smirnov test. Independent sample t test or non-parametric Wilcoxon was used to compare the differences of age, axial length and refractive results between the two groups. Pearson’s chi-square test was used for the investigation of the patient’s demographics and the accuracy between the two groups after the QCC application. P values lower than 0.05 were regarded as statistically significant.

## Results

### Patient demographics

Table 2 summarized the demographics of patients who underwent surgery before and after the QCC application. There were no significant differences in the sex ratio, age and axial length between the two groups.

**Table 2:** Demographics of cataract patients who underwent surgery before and after the quality control circle activity.

	Before QCC (eyes = 170)		After QCC (eyes = 151)		P value
	Mean ± SD	Range	Mean ± SD	Range	
Sex (M/F)	48/68		42/62		0.881
Age (yrs)	72.46 ± 11.55	41–95	70.36 ± 11.29	39–89	0.121
Axial length (mm)	25.22 ± 2.95	22.05–34.02	25.55 ± 2.99	22.24–33.8	0.273
<26 mm	23.62 ± 1.00	21.06–25.95	23.60 ± 0.79	22.24–25.41	0.98
≥26 mm	29.59 ± 2.09	26.26–34.02	29.25 ± 2.00	26.17–33.8	0.425

QCC = Quality control circle, SD = Standard deviation, F = Female, M = Male.

### Accuracy of IOL power estimation

Table 3 summarized the refractive outcomes of Haigis and SRK/T formulas in patients with different AL in the two groups. As shown in Table 4, there was significantly higher accuracy in patients with normal axial length after QCC when calculated by Haigis or SRK/T formulas, while there was no statistically significant difference in the patients with long axial length by the two regular formulas. However, we achieved a remarkable accuracy of IOL power prediction as high as 75.0% if the PhacoOptics software was used in patients with long AL, which was significantly higher than that using the other two formulas (p < 0.001).

**Table 3:** Refractive outcomes of Haigis and SRK/T formulas in patients with different axial lengths in the two groups.

Group	Without QCC group		QCC group		P value	
	ME	MAE	ME	MAE	ME	MAE
Normal AL						
Haigis	−0.09 ± 0.60	0.48 ± 0.38	−0.08 ± 0.44	0.31 ± 0.32	0.482	<0.001
SRK/T	−0.15 ± 0.59	0.49 ± 0.36	−0.02 ± 0.48	0.35 ± 0.33	0.064	<0.001
Long AL						
Haigis	−0.69 ± 0.99	0.86 ± 0.83	−0.75 ± 0.75	0.78 ± 0.73	0.5414	0.9211
SRK/T	−0.55 ± 1.03	0.80 ± 0.84	−0.61 ± 0.85	0.71 ± 0.78	0.6153	0.5803
PhacoOptics	—	—	−0.09 ± 0.69	0.45 ± 0.52	<0.001*	0.0012*
Total						
Haigis	−0.25 ± 0.77	0.58 ± 0.56	−0.31 ± 0.65	0.47 ± 0.54	0.6449	0.0058
SRK/T	−0.26 ± 0.75	0.57 ± 0.54	−0.22 ± 0.69	0.47 ± 0.55	0.5052	0.0081

QCC = Quality control circle, AL = Axial length, ME = Mean prediction error, MAE = Mean absolute prediction error. *The P value comes from comparing the results of PhacoOptics software with those of the Haigis and SRK/T formulas after the QCC activity.

**Table 4:** Accuracy of intraocular lens power prediction before and after the quality control circle activity.

	Within 0.5D			Within 1.0D		
	Without QCC	QCC	P value	Without QCC	QCC	P value
Normal AL						
Haigis	63.2%	80.8%	0.004	92.0%	97.0%	0.114
SRK/T	59.2%	75.8%	0.009	90.4%	93.9%	0.333
Long AL						
Haigis	40.0%	36.5%	0.886	73.3%	82.7%	0.264
SRK/T	44.4%	46.2%	0.726	80.0%	84.6%	0.551
PhacoOptics	—	75.0%	<0.001*	—	92.3%	0.315^*^

QCC = Quality control circle, AL = Axial length. *The P value comes from comparing the results of PhacoOptics with those of the Haigis and SRK/T formulas after the QCC activity.

### QCC protocol

**Figure 2:**
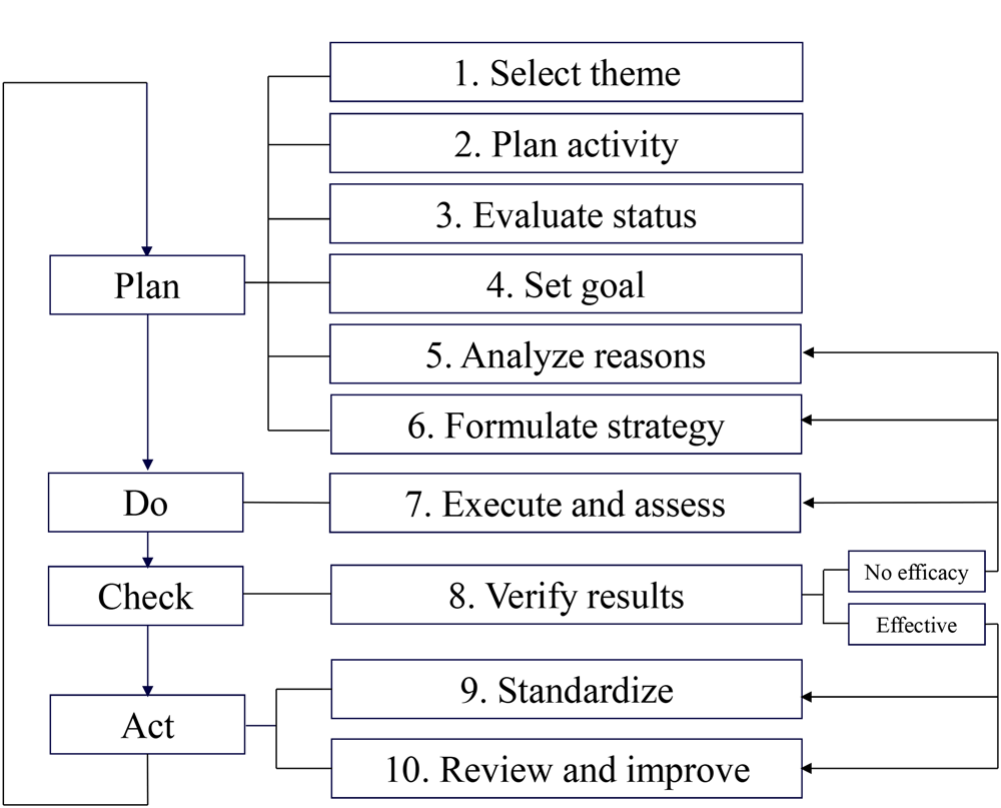
Protocol of the quality control circle. The ten steps and PDCA (plan, do, check and act) flow of the quality control circle.

We established the QCC group and selected the IOL power calculation accuracy improvement as our goal. Then after analyzing the current status and related reasons, we formulated several strategies and executed them. Finally, the results were checked and the process was standardized. During the QCC activity, the PDCA circulation would continue until the results were effective. The ten steps protocol of QCC was shown in Fig. 2, and the badge of our QCC could be found in Supplementary Fig. S1.

### Standardization

**Figure 3:**
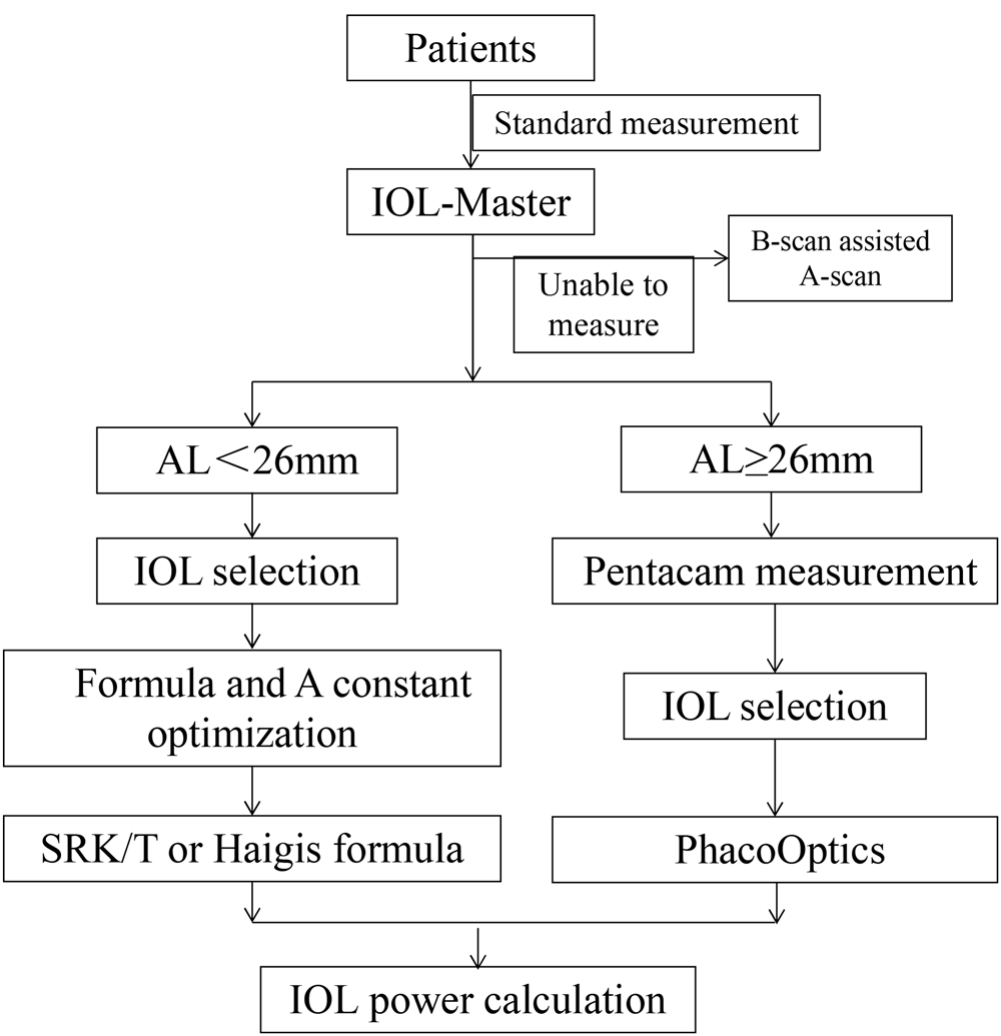
Standard flow of the intraocular lens (IOL) power calculation process. After quality control circle, the process of the IOL power calculation was standardized for cataract patients with different axial lengths (AL).

The standard operation flow (Fig. 3) was established and optimized by the continuous improvement of the QCC activity.

## Discussion

Cataract surgery is the only treatment for cataract patients at present, while accurate IOL power calculation continues to be a big challenge in ophthalmology, especially in primary hospitals^1–8,22–24^. However, unoptimized process of IOL power calculation may lead to inaccurate refractive outcomes. Process optimization has drawn great attention worldwide for its potential in accuracy improvement^23–26^. Nevertheless, QCC, as a process optimization tool, has never been used in the field of ophthalmology. Therefore, we firstly performed this study to optimize the IOL power calculation process by utilizing QCC technique and evaluated its efficacy in improving the accuracy of IOL power calculation.

Through our retrospective data analyses, we found that before QCC, the percentages of eyes within 0.5D of target refraction were respectively 63.2%/59.2% (Haigis/SRK/T formula), which were consistent with the benchmark standards^8^. Although they were lower than other literature^3–5^, we thought they were acceptable as the baseline since they were obtained under conditions with unoptimized process including biometric measurement, A constant and formula selection. Based on the QCC technique, our study analyzed the potential reasons related to the low accuracy by data review and brainstorm. In order to optimize the process and achieve a higher level of accuracy of IOL power estimation, we laid down relevant solutions to realize this goal.

Given that feasibility, economy and circle capacity, three leading causes were selected as the main target (see Supplementary Table S1). In China or other third world countries, patients are more prone to undergo the cataract surgery until the cataract is mature, thus A scan or other enhanced optical measure methods will be required. Therefore, accurate biometric measurement and standardized process are essential and critical. For the purpose of more precise biometric measurement, we formulated the standard operation procedure such as using the average of repeated measurement and/or checking the results by different operators, especially in patients performed by A scan. In fact, no matter how well we controlled the protocol, the precision of the A scan is inferior to the optical measurement, and the axial length performed by A scan will influence the IOL power calculation more or less. In terms of IOL^23^, we optimized our routine IOL type, A constant and formula by refer to the ULIB. With respect to formula selection in patients with abnormal axial length especially long axial length, we found that researchers have introduced the C constant as a new concept for the ray-tracing assisted IOL calculation software PhacoOptics in the latest literature, which indeed bettered the prediction of the IOL power^20,21^. Therefore, we applied this method in IOL power calculation in patients with long axial length. It turned out that the accuracy was 75%, which was consistent with other literature^3,5,6,22,23,26^.

In accordance with the PDCA cycle of the QCC activity, the accuracy of predicted IOL power increased from 63.2%/59.2% (Haigis/SRK/T formula) to 80.8%/75.8% respectively in patients with normal axial length and the results were similar with other studies^3,6,22^. Although there was no difference between the two formulas after QCC in patients with long axial length, the utilization of PhacoOptics software led to a dramatic growth in accuracy. In patients with normal axial length, we attributed the enhancement of accuracy to the standardization of axial length measurement, IOL constant and formula optimization, indicating that the whole process optimization could lead to accuracy improvement. Regarding to the IOL power calculation in patients with long axial length, both the Haigis and SRK/T formulas would take the axial length and anterior chamber depth into consideration, while the PhacoOptics software was independent of axial length as it was based on the preoperative lens thickness and anterior chamber depth to predict the postoperative IOL position^20,21^. In addition, as an optical formula, PhacoOptics software attached great importance to the true net corneal power, realistic geometric position and corneal asphericity. Furthermore, previous study has demonstrated that C constant was an unbiased concept to predict the effective lens position^20^. Hence, we owed the success in patients with long axial length to the ray tracing method. Although the ray tracing method demonstrated its potential and efficacy in accuracy improvement, we did not apply it in patients with normal AL because of the desirable accuracy by Haigis/SRK/T formula, high manpower costs and cost-benefit analysis. In this study, our department utilized the scientific management approach as a tool, established the QCC group, executed the strategies under the PDCA principle, and obtained desirable results, which was a pioneering attempt and validated the importance of QCC in ophthalmology. It not only gave rise to accuracy, but also promoted the formation of standardization and a smooth process flow. Furthermore, it increased the satisfaction of patients as well. As we know, the worsening of doctor-patient relationship is becoming an increasing threat in China due to misunderstanding and distrust^27,28^. The dramatic growth in accuracy could bring relief in the deteriorated condition and facilitate the harmonious coexistence between patients and doctors.

Promoting the QCC activity can not only obtain the tangible achievements such as accuracy improvement, but also produce some intangible achievements. First, the QCC activity could draw out the capacity and potential ability of the group. Circle members are willing to spend more time, strength and creativity to realize the self-management and harmonious development of the group. In the modern society, the pattern of solving problems by a single department is no longer an ideal method. Additionally, allowing for the involvement of the QCC activity with many different departments, a sense of responsibility and coordination will be promoted. Overall, the more active the QCC is, the better results we can achieve, and everyone can benefit from the process. Moreover, the QCC activity exerted some certain effect to encourage the work morale and attitudes. Although this investigation only performed in cataract patients, we hope that our novel discovery will bring more studies to further explore the function of QCC in ophthalmology.

In spite of the success in this study, several limitations should also be noted. First, a large and multi-center study was definitively needed to verify the efficacy and accuracy of QCC. In addition, as limited by economy and circle capacity, abnormal anterior chamber depth modification was not performed in the present study. Thus, further investigations were still required for the benefits of complex cases.

In summary, this study showed the potentially important role that optimization of process has in the IOL power calculation in patients who underwent cataract surgery. The results proved the efficacy and accuracy of QCC in IOL power calculation process optimization and accuracy improvement. The application of QCC really made a difference from the conventional methods, indicating the feasibility of extended utilization of QCC in more fields of ophthalmology, clinical practice and even the scientific research. Furthermore, the successful experience of QCC could be beneficial to the establishment of primary eye hospitals. With the current medical climate of precision medicine and personalized medicine^10,11,28^, there is no doubt that QCC should be considered as a promising approach to achieve a higher level of efficacy and precision in medical and research field.

### Meeting

This study was presented at the Annual Meeting of the Association for Research in Vision and Ophthalmology (ARVO) during May 7 to May 11, 2017 in Baltimore, Maryland, and selected as a hot topic.

## Electronic supplementary material

Supplementary Fig. S1

## References

[1] Olsen T (2007). Calculation of intraocular lens power: a review. Acta ophthalmologica Scandinavica.

[2] Gale RP, Saldana M, Johnston RL, Zuberbuhler B, McKibbin M (2009). Benchmark standards for refractive outcomes after NHS cataract surgery. Eye (London, England).

[3] Kane JX, Van Heerden A, Atik A, Petsoglou C (2017). Accuracy of 3 new methods for intraocular lens power selection. Journal of cataract and refractive surgery.

[4] Cooke DL, Cooke TL (2016). Comparison of 9 intraocular lens power calculation formulas. Journal of cataract and refractive surgery.

[5] Aristodemou P, Knox Cartwright NE, Sparrow JM, Johnston RL (2011). Formula choice: Hoffer Q, Holladay 1, or SRK/T and refractive outcomes in 8108 eyes after cataract surgery with biometry by partial coherence interferometry. Journal of cataract and refractive surgery.

[6] Wang L, Shirayama M, Ma XJ, Kohnen T, Koch DD (2011). Optimizing intraocular lens power calculations in eyes with axial lengths above 25.0 mm. Journal of cataract and refractive surgery.

[7] Arnedos M (2015). Precision medicine for metastatic breast cancer–limitations and solutions. Nature reviews. Clinical oncology.

[8] Collins FS, Varmus H (2015). A new initiative on precision medicine. The New England journal of medicine.

[9] de Bono JS, Ashworth A (2010). Translating cancer research into targeted therapeutics. Nature.

[10] Hill DC (2017). Intraoperative aberrometry versus preoperative biometry for intraocular lens power selection in axial myopia. Journal of cataract and refractive surgery.

[11] Hoffer KJ (2015). Protocols for studies of intraocular lens formula accuracy. American journal of ophthalmology.

[12] Karpel J, Spencer R, Schamberger T, Klein W (1983). Record ring: moving toward solutions–a quality control circle in a medical record department. Journal (American Medical Record Association).

[13] Matsuda K (1983). Definition of the QC (quality control) circle activities. Kango tenbo. The Japanese journal of nursing science.

[14] Matsuda K (1983). Initiation of QC circle and problem-solving technique. Kango tenbo. The Japanese journal of nursing science.

[15] Munchus G (1983). Employer-employee based quality circles in Japan: Human resource policy implications for American firms. Academy of Management Review.

[16] Krause, G., Benzler, J., Reiprich, G. & Görgen, R. Improvement of a national public health surveillance system through use of a quality circle (2006). 17206025

[17] Chen P (2016). Role of quality control circle in sustained improvement of hand hygiene compliance: an observational study in a stomatology hospital in Shandong, China. Antimicrobial resistance and infection control.

[18] Zhu LL, Li W, Song P, Zhou Q (2014). Injection device-related risk management toward safe administration of medications: experience in a university teaching hospital in. The People’s Republic of China. Therapeutics and clinical risk management.

[19] Forster DH (2000). Can quality circles improve hospital-acquired infection control?. The Journal of hospital infection.

[20] Olsen T, Hoffmann P (2014). C constant: new concept for ray tracing-assisted intraocular lens power calculation. Journal of cataract and refractive surgery.

[21] Olsen T, Funding M (2012). Ray-tracing analysis of intraocular lens power *in situ*. Journal of cataract and refractive surgery.

[22] Abulafia A (2015). Intraocular lens power calculation for eyes with an axial length greater than 26.0 mm: comparison of formulas and methods. Journal of cataract and refractive surgery.

[23] Aristodemou P, Knox Cartwright NE, Sparrow JM, Johnston RL (2011). Intraocular lens formula constant optimization and partial coherence interferometry biometry: Refractive outcomes in 8108 eyes after cataract surgery. Journal of cataract and refractive surgery.

[24] Wang JK, Hu CY, Chang SW (2008). Intraocular lens power calculation using the IOLMaster and various formulas in eyes with long axial length. Journal of cataract and refractive surgery.

[25] Zhao, D. *et al*. Optimizing glaucoma screening in high risk population: design and 1-year findings of the Screening to Prevent (SToP) Glaucoma study. American journal of ophthalmology, 10.1016/j.ajo.2017.05.017 (2017). 10.1016/j.ajo.2017.05.017 28549849

[26] Petermeier K (2009). Intraocular lens power calculation and optimized constants for highly myopic eyes. Journal of cataract and refractive surgery.

[27] Huang J, Yan L, Zeng Y (2010). Facing up to the threat in China. Lancet (London, England).

[28] Zeng J, Zeng XX, Tu Q (2013). A gloomy future for medical students in China. Lancet (London, England).

