# Supplementary material for: Sustained accuracy improvement in intraocular lens power calculation with the application of quality control circle
Source: Sci Rep. 2017 Nov 1;7:14852. doi: 10.1038/s41598-017-14171-9 (PMC5665969; doi:10.1038/s41598-017-14171-9)
Supplement: Supplementary file 1 — Supplementary Fig. S1 [file 41598_2017_14171_MOESM1_ESM.pdf]

Title page

Title: Sustained accuracy improvement in intraocular lens power calculation with the application of quality control circle

Authors List:

Lei Lin[1]; Pingjun Chang[1]; Jialu Xie[1]; Zhangliang Li[1]; Hongfang Zhang[1]; Fan Lu[1*]; Yun-e Zhao[1*]

1. School of Ophthalmology and Optometry, Wenzhou Medical University, Wenzhou, Zhejiang, China

Corresponding author:

Yun-e Zhao and Fan Lu contributed equally to this study.

Yun-e Zhao. E-mail: zye@mail.eye.ac.cn; zyehzeye@126.com

Fan Lu. E-mail: lufan62@mail.eye.ac.cn

Address for reprints:

School of Ophthalmology and Optometry, Wenzhou Medical University, Xueyuan Road 270, Wenzhou, Zhejiang, China. ZIP: 325102.

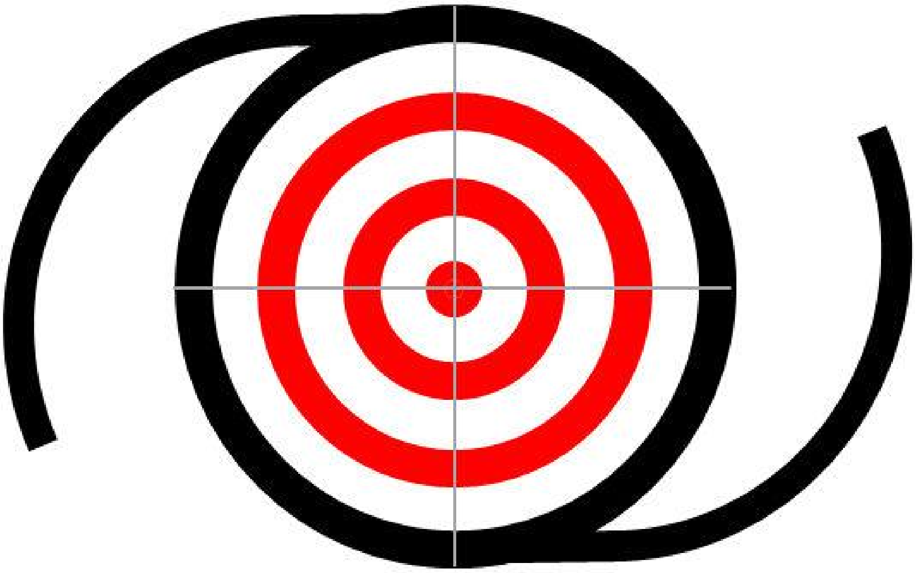

Supplementary Fig. S1 Quality control circle badge. An intraocular lens (IOL) sketch consists of the badge frame and a series of red circles composing the IOL optical zone. A grey cross intersects with the center of the red target, indicating the target of our circle was to optimize the IOL and promote the accuracy of refractive power prediction after cataract surgery.
